# Invasive pneumococcal surveillance to assess the potential benefits of extended spectrum conjugate vaccines (PCV15/PCV20) in older adults

**DOI:** 10.1017/S0950268823000110

**Published:** 2023-01-26

**Authors:** Hilary Humphreys, Mary Corcoran, Jolita Mereckiene, Robert Cunney, Suzanne Cotter

**Affiliations:** 1 Department of Clinical Microbiology, Royal College of Surgeons in Ireland University of Medicine and Health Sciences, Dublin, Ireland; 2 Irish Meningitis and Sepsis Reference Laboratory, Children's Health Ireland at Temple Street, Dublin, Ireland; 3 Department of Microbiology, Children's Health Ireland at Temple Street, Dublin, Ireland; 4 Health Services Executive – Health Protection Surveillance Centre, Dublin, Ireland

**Keywords:** Conjugated vaccines, elderly, invasive pneumococcal disease, PCV13, PCV15, PCV20, PPV23, serotypes, strategy

## Abstract

The introduction of pneumococcal conjugate vaccines (PCV) into the childhood vaccination programme has reduced invasive pneumococcal disease (IPD). Although anticipated from data elsewhere, surveillance in Ireland has confirmed reductions in IPD amongst those ⩾65 years of age due to a decline of PCV serotypes in this age group. Currently, direct protection against IPD in the elderly is focused on immunisation with the 23-valent pneumococcal polysaccharide vaccine (PPV23). However, immunity may not be as effective as with PCV and, furthermore, PPV23 uptake is poor in Ireland. Hence, consideration should be given to providing a PCV to this age group.

## Introduction

*Streptococcus pneumoniae* is a major cause of life-threatening infections such as meningitis and bloodstream infection, i.e. invasive pneumococcal disease (IPD). The population groups at highest risk of pneumococcal infection are young children and adults ⩾65 years of age. Even before the success of pneumococcal vaccines in children, the Centers for Disease Control and Prevention (CDC) in the USA estimated that the mortality rate due to IPD was much greater in adults ⩾65 years of age (18/100 000 population) than in children <2 years (0.4/100 000) in the post vaccine-era [1].

*S. pneumoniae* is a successful pathogen, in part due to the diversity of the circulating capsular serotypes, with up to 100 immunologically distinct serotypes identified [2]. Conjugate vaccines were developed to reduce the burden of the predominant serotypes circulating in paediatric populations at the time of development by eliciting an antibody response to the capsule polysaccharides of the pneumococcus of the vaccine-targeted serotypes. However, serotype prevalence varies depending on population demographics, vaccination strategies, vaccine uptake and other factors. While adult pneumococcal vaccination programmes have an impact on non-bacteraemic community-acquired pneumonia due to *S. pneumoniae*, this is often not captured by national surveillance programmes. Here, we describe the laboratory surveillance data of IPD in Ireland since 2008 with extensive characterisation and serotyping of isolates to track patterns of disease and to inform national vaccination strategy [3, 4].

In September 2008, PCV7 was introduced to the Irish infant immunisation schedule at 2, 6 and 12 months of age, offering protection against seven serotypes, i.e. 4, 6B, 9V, 14, 18C, 19F and 23F which were commonly associated with invasive disease in children at that time. In December 2010, PCV7 was replaced by the 13-valent pneumococcal conjugate vaccine (PCV13), which offers protection against six additional serotypes; 1, 3, 5, 6A, 7F and 19A. In December 2016, the schedule for children born on or after 1 October 2016 was changed to a dose of PCV13 at 2, 6 and 13 months. This was done to accommodate vaccination with the newly introduced *Neisseria meningitidis* type B vaccine at 12 months. The uptake of the three doses of PCV13 at 24 months of age remains high in Ireland at 84.5% (ranges 81–92%) [5], despite challenges created with the coronavirus disease 2019 (COVID-19) pandemic. More recently, two new vaccines (PCV15 and PCV20) have been approved for use in adults by the European Medicines Agency (EMA) [6, 7]. While not part of the current vaccine schedule in Ireland, we consider the additional serotype coverage and potential protection provided by these broader spectrum vaccines, whilst acknowledging that with new vaccines, there may be lower immune responses to some of the serotypes, e.g. serotype 8, than with existing PCVs (https://www.cdc.gov/vaccines/acip/recs/grade/pneumo-PCV20-risk-based-etr.html).

A 23-valent polysaccharide vaccine (PPV23) is currently recommended for adults ⩾65 or for high-risk adults <65 years with immunosuppressive conditions or co-morbidities. PPV 23 vaccine is available since 1996 in Ireland (https://www.hse.ie/eng/health/immunisation/hcpinfo/guidelines/chapter16.pdf (accessed 19-10-2022).

However, uptake in adults in Ireland is low in comparison to the paediatric schedule (27–36% vs 81–92%), as pneumococcal vaccination is part of the routine childhood vaccination programme whereas in adults access is more opportunist and it is not always free. Furthermore, it has limited effectiveness against non-bacteraemic community-acquired pneumonia. Therefore with this variable low uptake, it is difficult to assess any impact this may have had on serotype epidemiology in Ireland [3, 8]. Since 2015, a single dose of PCV13 prior to PPV23 administration is recommended for those with immunosuppressive conditions or co-morbidities. National pneumococcal vaccination guidance is available in http://www.hse.ie/eng/health/immunisation/hcpinfo/guidelines/chapter3.pdf (accessed 19-10-2022).

## IPD in the elderly and serotype distribution

**Fig. 1.**
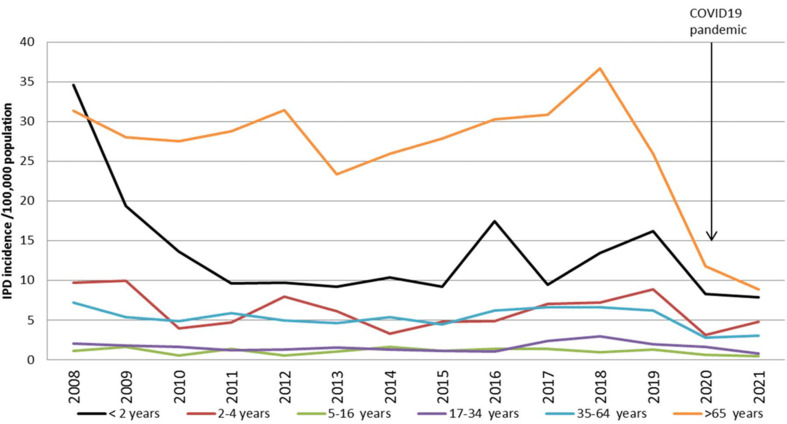
Incidence rate of invasive pneumococcal disease according to patient age based on isolates typed, from 2008–2021 (data source: Health Protection Surveillance Centre; data correct as of September 2022).

In Ireland, adults ⩾65 years of age remain at highest risk of IPD with an incidence rate (IR) of 8.89/100 000 in 2021 (Fig. 1). This is lower than in previous years, which peaked in 2018 (IR = 36.68/100 000) and during the pre-pandemic period of 2019 (IR = 26.00/100 000). The overall decline of IPD was also observed in most countries globally and is likely to be associated with a number of containment factors associated with reducing the spread of respiratory illnesses during the COVID-19 pandemic [9].

With the uptake of PPV23 markedly low in Ireland (27–36%), it is unlikely that vaccination impacted on the serotype epidemiology [3, 8]. Similar to results in children, the number of PCV7 cases dropped after the vaccine was introduced to the paediatric schedule, which was likely to be associated with a decrease in carriage and herd immunity [3, 4]. While PCV13 cases have declined in recent years, two predominant PCV13 serotypes persisted in 2020 and 2021, and with contrasting trends. Serotype 19A represented 15% and 7% of cases in older adults in 2020 and 2021, respectively, while serotype 3 increased from 7% to 12% in 2020 and 2021, respectively. Direct vaccination with a conjugate vaccine that includes these serotypes, rather than reliance on herd-protection alone may potentially reduce the incidence of these PCV included serotypes, but the post-licensing studies are not unanimous [10, 11]. Aside from those serotypes most other IPD cases were associated with non-PCV13 serotypes. During the pandemic years when there was social distancing and a greater emphasis on infection prevention measures, this impacted on the epidemiology of many other infections. Nonetheless, the predominant non-PCV13 serotypes in adults ⩾65 years of age in 2020 and 2021 included serotype 22F (7% and 8% of cases in 2020 and 2021, respectively) which is covered in PCV15 and PCV20, serotype 8 (28%, 12%) which is covered in PCV20, and 35B (5%, 9%) which is not covered in any of the PCV's. Serotype 23B, which is of concern as it is not covered in the two recently approved vaccines, PCV15 nor PCV20, also increased from 1% in 2020 to 6% of cases in 2021 in older adults [12]. This may be of further concern in future years when the impact of COVID-19 pandemic measures have waned. These serotypes may persist as there was also an increase among children, along with an increase in antimicrobial resistance associated with this serotype (data not included in this report).

**Fig. 2.**
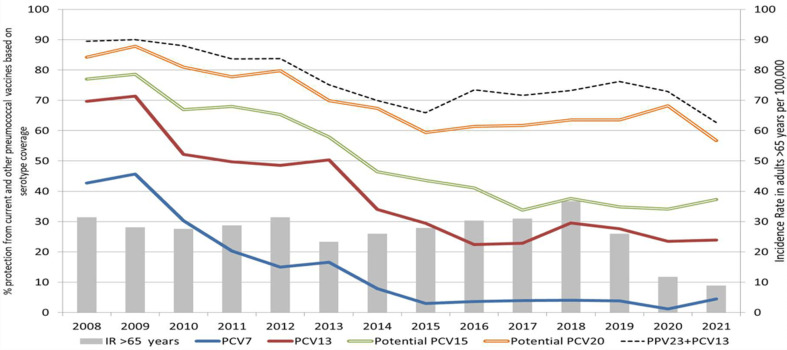
Protection for adults ⩾65 years of age provided by vaccines based on the proportion of invasive pneumococcal disease caused by a vaccine serotypes categories (PCV7, PCV13, PCV15, PCV20, PPV23 + PCV13), from 2008 to 2021. Serotypes included in each vaccine category were all 13 serotypes in PCV13, all 15 serotypes included in PCV15 and all 20 serotypes in PCV20. There is some overlap in the overall coverage, but it is reflective of total coverage of each PCV type. PPV23 coverage is inclusive of one dose of PCV13 and one dose of PPV23. Neither PCV15 nor PCV20 are currently available in Ireland.

As displayed in Figure 2 increased vaccination uptake (PPV23) or changes in the vaccine schedule (to include PCV15 or PCV20) could provide protection against IPD serotypes associated with older adults. In 2021, 37%, 57% and 63% of IPD cases were caused by serotypes covered in PCV15, PCV20 and PPV23, respectively. While polysaccharide vaccines are not reported to provide as effective immune response in older adults in comparison to conjugate vaccines (PCVs) [13, 14], PPV23 is still retained after a single dose of PCV13 but not if PCV20 is used [14]. These recommendations have now changed to include PCV15 and PCV20 [15]. In Ireland, a single dose of PCV13 prior to PPV23 administration is recommended only for those adults with immunosuppressive conditions or co-morbidities. However, based on the IPD data from 2021, between 37% and 57% of IPD cases in older adults were PCV15 or PCV20 vaccine preventable. Therefore direct immunisation, rather than merely relying on herd protection from PCV13 in children could reduce the burden of disease in this older population. While PPV23 serotype coverage was also high in this population (63% of PPV23 serotypes in 2021 in those ⩾65 years of age), with low vaccine uptake in Ireland and waning immunity reported elsewhere [13], direct vaccination with a PCV may provide greater protection to older adults who now bear the highest disease burden. There may also be a case for including one of the new PCVs (PCV15 or PCV20) in the immunisation of older people and immunosuppressed adults, with or without the continued use of PPV23 in the same age categories [16].

## Impact of vaccination in the elderly

A number of publications have documented the effectiveness of PCV on IPD across all ages, especially in children, but with benefits also accruing to the elderly [16, 17]. A multi-centre study in 10 countries across 13 sites assessed over five years the indirect effects in older adults (⩾65 years) and found that there was a 9% reduction in all serotypes and a 38% reduction in PCV7 and additional PCV 13 serotypes [18]. However, there were some increases in non-PCV13 episodes of IPD. A public health evaluation of a clinical trial of PCV13 in adults conducted post-hoc found substantial reductions in IPD and community-acquired pneumonia caused by *S. pneumoniae* [19]. Hence, conjugate vaccination has benefits in the elderly when administered only as part of childhood vaccination and when also administered to the adult population. However, some serotypes such as 3 and 19A still remain persistently associated with IPD in older adults in Ireland.

Pneumococcal vaccination is routine in many countries for the elderly, but usually still with the polysaccharide vaccine. A review of the CDC recommendations for the use of PCV15 or PCV20 before changing guidelines found cost savings in all scenarios for use of either PCV20 alone or PCV15 in series with PPSV23 for all adults aged ⩾65 years [15]. The economic model for PCV20 alone for all adults aged ⩾65 years found estimated cost savings of up to $ 39 000 per quality-adjusted life-year (QALY) gained, while for PCV15 in series with PPSV23 for all adults aged ⩾65 years, the estimated the cost savings were up to $ 282 000 per QALY gained [15]. Given the overall rate of some PCV13-serotypes is much higher in Ireland [3] and other European countries [18] in comparison to the US, it is likely that direct vaccination with a PCV could offer even more benefits in European countries, particularly those with persistence of serotype 3 and 19A.

## Surveillance and future vaccination strategy

In conclusion, continued national surveillance of serotypes causing IPD is necessary to: monitor epidemiology, assess the effectiveness of the national vaccination programme, detect the presence of non-vaccine serotypes, and monitor the emergence of replacement serotypes. For example, genomics has revealed the emergence of a sub-clade unique to Ireland which included five of ten vaccine failures, possibly indicating alterations in capsular polysaccharide biosynthesis that impact on bacterial persistence [20].

The introduction of the PCV7/13 in Ireland has resulted in a reduction in the number of IPD cases. However, it is important to note that these new vaccines together with PPV23 could still reduce the burden of disease in older adults further as PCV15 and PCV20 serotypes still account for 37-57% of cases in this patient group. Hence each country needs to decide its strategy for protecting older adults based their national epidemiology. These may comprise (a) PPV23 only, (b) PCV followed by PPV23 for all, (c) PCV followed by PPV23 only for those with immunosuppression or (d) PCV (13, 15 or 20) for all.

## Data Availability

IPD is a notifiable disease in Ireland and all epidemiological and laboratory data was collected to inform public health advice and strategy. Most if not all of the aggregated data is available publicly but we are happy to facilitate reasonable requests from researchers and others in accessing other data on request.
